# Selection with support: How cross-modal attention shapes smooth pursuit eye movements

**DOI:** 10.1007/s00426-026-02328-z

**Published:** 2026-06-10

**Authors:** Geoffrey Kaye, Adam C. Ricci, Ella Weaire, Zarnaab Syed, Natalie Gasson, Emily J. Corti, Welber Marinovic

**Affiliations:** School of Population Health, Faculty of Health Sciences, Curtin University, Perth, Australia

## Abstract

Smooth pursuit eye movements can offer a window into the interaction between sensorimotor control and cognition. The present study examined how pursuit might be affected by dual-task demands varying in sensory modality and cross-modal conflict. A second, exploratory aim was to assess whether individual differences in working memory (WM) capacity could moderate these effects. Participants were asked to track a moving target whilst performing mental calculations on visual, auditory, or bimodal digit streams. Pursuit was stabilised when attention was directed to visual input but substantially destabilised when directed to auditory input, revealing a bidirectional modality asymmetry. When attention was focused on an auditory stream in the presence of incongruent visual digits, cognitive error increased whilst pursuit variability did not, suggesting that additional conflict may tax cognitive rather than oculomotor processes. Higher WM capacity appeared to provide a modest, selective buffer, reducing pursuit variability specifically under demanding auditory conditions. We interpret these findings through a Selection-with-Support (SWS) principle: dual-task costs depend upon the representational alignment between attentional selection and the motor circuits supporting pursuit. Visual attention appears to directly support pursuit by engaging shared sensorimotor circuits, whilst auditory attention, lacking such premotor grounding, may rely upon executive control that WM can only partially sustain. The present study therefore offers a plausible mechanistic account of how attention might be motorically grounded.

## Public Significance Statement

This study looked at how eye movements during the tracking of moving objects are affected when people also do mental tasks. Tracking remained consistent when attention was on what people saw, but became variable when attention shifted to what they heard. The findings suggest that paying attention to visual information is closely tied to the brain systems that control eye movement.

## Introduction

Smooth pursuit eye movements (SPEM) provide a unique window into the interplay between cognitive and motor systems. The successful tracking of a moving target demands more than a simple reflex action; it is dependent on the integration of sensory prediction, motor coordination, and cognitive control. This seemingly simple movement would require the continuous transformation of ever-changing visual inputs into precise motor commands, a process actively supported by cognitive functions such as sustained attention and working memory (WM) (Hutton & Tegally, 2005). Not surprisingly, given the complexity of this system, even subtle impairments in SPEM become evident when measured with sensitive tracking devices. Such impairments are believed to serve as possible biomarkers for cognitive dysfunction in a number of clinical populations, including people with schizophrenia (Brakemeier et al., 2020; Morita et al., 2020) and mild traumatic brain injury (mTBI; Stubbs et al., 2019). This phenomenon has motivated several studies employing dual-task paradigms to study how the synchronous taxing of neural resources affects oculomotor control and executive function. The present study extends this line of investigation by examining how pursuit interacts with cognitive load and cross-modal interference, with an exploratory focus on whether individual differences in WM capacity modulate these effects.

To understand these interactions, it is important to consider how cognitive processes shape pursuit behaviour. Several studies have demonstrated that eye-movements, including smooth-pursuit, can be affected by cognitive processes. For example, evidence from oculomotor delayed-response tasks showed that WM for visual motion can support predictive SPEM (Fukushima et al., 2013). More specifically, using single neuron recording in behaving monkeys, Fukushima and colleagues showed that sustained activity in the supplementary eye fields (SEF), prefrontal cortex, and cerebellum during memory delays is consistent with a role in storing motion information for subsequent movement preparation. When these areas are compromised, as in Parkinson’s disease, predictive pursuit deficits become evident, demonstrating the importance of executive function for oculomotor control (Fukushima et al., 2013). In addition, other behavioural studies have shown that SPEM benefits from memory of prior target motion: when stimulus histories are consistent, predictive anticipation improves and tracking errors decrease (Miyamoto et al., 2021). Interestingly, this occurs both when target motion is predictable, and is also observed when target motion is unpredictable, suggesting that pursuit control may rely on predictions based on the weighted integration of recent sensory and motor experiences, as shown for example in visuomotor prediction tasks (de Rugy et al., 2012). Taken together, these studies indicate that pursuit is shaped by the contents of WM as well as by recent sensory experiences that support predictive control. However less is known about how concurrent cognitive demands influence this control.

Complementing this work on predictive mechanisms, dual-task paradigms have provided evidence that SPEM is affected by WM load. However, the nature of this interaction remains unclear. Some studies have reported that higher WM load can improve pursuit consistency. For instance, Stubbs et al. (2019) found that performing a concurrent 1-back task reduced SPEM variability in both healthy controls and people with mTBI, but when the working memory load increased (2-back), only the control group showed a further reduction in variability. These results suggest that in healthy participants, WM engagement may enhance pursuit by focusing attention on the target. However, the benefits of WM engagement appear to depend on the specific nature and modality of the secondary task. This is evident from the work of Contreras et al. (2011) who showed that when required to engage in an auditory memory task, people with mTBI displayed increased SPEM variability, whereas control participants improved tracking under intermediate levels of cognitive load.

These divergent findings may reflect different types of resource overlap between SPEM and WM. Research on visual working memory has demonstrated reciprocal interactions with attentional control during dynamic visual tasks. For example, Makovski and Jiang (2009) showed that maintaining perceptual surface features of a target, such as colour, in WM facilitates the tracking of moving objects, indicating that WM can support attentional selection during motion. In contrast, when WM is heavily loaded, tracking performance declines, revealing competition for shared cognitive resources. Similarly, Yue et al. (2017) found that holding colours in WM interfered with smooth pursuit, and conversely, pursuit direction influenced WM accuracy. Altogether, the literature reviewed here seems to suggest that pursuit and WM share overlapping attentional resources that can either cooperate or compete depending on task demands.

Recent work by our group sought to reconcile some of these discrepancies by directly comparing visual and auditory dual-task conditions during SPEM (Kaye et al., 2025). We demonstrated a modality-specific asymmetry: a secondary visual task reduced tracking variability, whereas an auditory task of equivalent difficulty increased it. This finding suggests that pursuit interference does not simply depend on a common pool of cognitive resources drawn as a function of load but is sensitive to the alignment of sensory and attentional modalities. We proposed that auditory processing may require greater cognitive effort to generate and maintain internal representations, potentially through visual imagery, which competes with resources required for smooth pursuit control. In contrast, visually presented information may engage oculomotor circuits with comparatively little executive mediation. However, that study employed relatively straightforward dual-task conditions, leaving unresolved whether these effects hold under higher cognitive demands. To address this gap, the present study was designed to manipulate the unimodal and bimodal streams of digits while directing attention to either auditory or visual input during SPEM. In addition, we measured individual differences in WM capacity to determine whether it can serve as a general performance enhancer or as a selective buffer against modality-specific interference. We hypothesised that pursuit would be most vulnerable when attention must be sustained in an orthogonal modality, and that individuals with higher WM capacity would show greater resistance to such cross-modal interference.

## Method

### Participants

Forty young, healthy adults (26 female) aged between 18 and 31 years (M = 20.78, SD = 2.72) participated in this study in exchange for course credit. Participants were recruited from the undergraduate psychology program at the Curtin School of Population Health and reported normal or corrected-to-normal vision with no history of neurological conditions affecting eyesight. No formal acuity test was performed but we asked participants if they could see the numbers during familiarisation trials. The sample size was determined based on our previous study (Kaye et al., 2025), which employed a similar smooth pursuit paradigm using an identical cognitive load secondary task. A post hoc power simulation on that study’s fitted linear mixed model (using the simr package in R) indicated that the primary Condition × Modality interaction achieved approximately 80% power at *n* = 8 and virtually 100% power at *n* = 34. We increased the sample to 40 participants to accommodate the additional conditions and the inclusion of a between-participant moderator (working memory capacity) in the present design. All participants provided written informed consent prior to participation. Ethical approval was obtained from the Curtin Human Research Ethics Committee (HRE2018-0257). More details on the apparatus, stimuli, task, and analyses can be found in Kaye et al. (2025).

### Apparatus

Briefly, visual stimuli were displayed on a BenQ XL2420TE monitor (24-inch, 1920 × 1080 resolution, 60 Hz), auditory stimuli were delivered via headphones (Corsair, HS55 Stereo), and eye position was recorded using an EyeLink 1000 Plus (SR Research, Ontario, Canada) sampling at 1000 Hz. The experiment was programmed using MATLAB 2015b with PsychToolbox extensions (Brainard, 1997; Kleiner et al., 2007). Data from the right eye were analysed.

### Stimuli and task

**Fig. 1:**
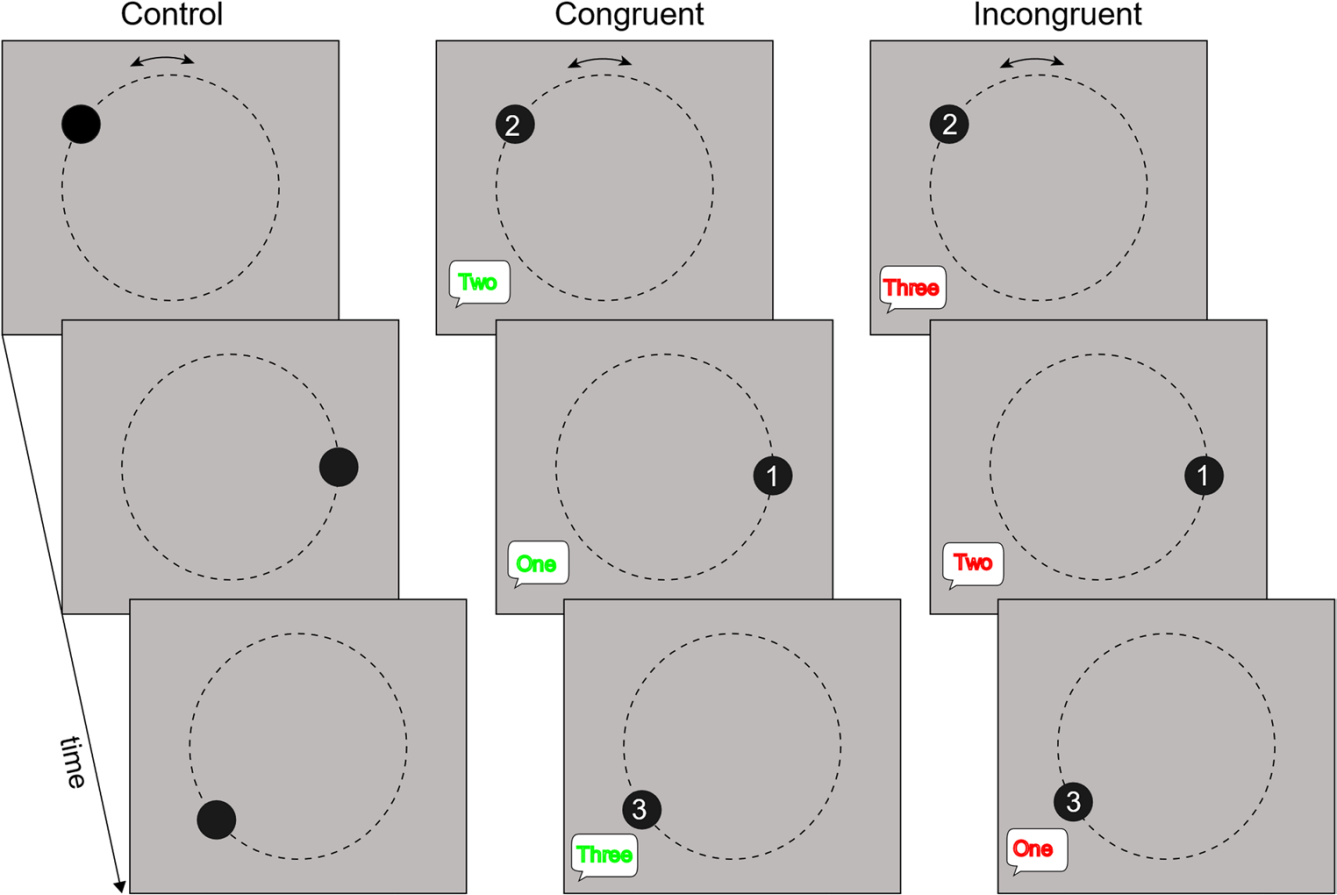
Illustration of the smooth pursuit eye movement (SPEM) task and examples of stimuli for three of the six experimental conditions: control, bimodal congruent, and bimodal incongruent. The primary task required participants to track a black circle moving at a constant velocity along a circular trajectory. The dashed line, representing the target’s trajectory, was not visible to participants. The Control condition (Left) involved only the SPEM task with no concurrent numbers presented. The Bimodal Congruent condition (Middle) presented identical, congruent numbers simultaneously both visually (within the pursuit target, e.g., “2”) and auditorily (represented by the green speech bubble “Two”). The Bimodal Incongruent condition (Right) illustrates an incongruent trial where different numbers were presented visually (e.g., “2”) and auditorily (represented by the red speech bubble “Three”). In all non-control conditions, participants performed a concurrent arithmetic task, continuously updating a running total by adding a random number (1–5) to the number presented in the cued modality (visual or auditory). Unisensory conditions are not shown in this figure

The primary task involved smooth pursuit eye movements in which participants tracked a black circle (subtending 1.8° of visual angle) on a grey background (see Fig. 1). The pursuit target moved along a circular trajectory with a radius of 9.2° of visual angle, with a period of 2.5 s (0.4 Hz), resulting in a constant tangential velocity of 26°/s, following Stubbs et al. (2018) and Kaye et al. (2025). The direction of the target’s motion (clockwise or counterclockwise) was randomly assigned on a trial-by-trial basis.

Concurrent with the tracking task, participants performed an arithmetic task in which numbers were presented either visually, auditorily, or bimodally. Numbers had a height of 0.6° of visual angle and were displayed every 1.5 s, resulting in a total of 17 numbers per pursuit trial. The first number in the sequence appeared 5 s after the onset of the pursuit target’s motion. In all conditions except the control, a random number between 1 and 5 was presented. Participants were instructed to mentally add this number to the previously presented number, updating the running total continuously. In the visual condition, numbers were presented within the central disc of the moving pursuit target. In the auditory condition, numbers were delivered through headphones at a peak intensity of approximately 65 dB(A). Each number was presented for 500 ms, with auditory stimuli temporally adjusted using Audacity software to match the duration of visual presentations.

The experimental design included six conditions. In the control condition, no numbers were presented during pursuit. In the bimodal congruent condition, identical numbers were presented simultaneously in both modalities, serving as a second baseline. The remaining four conditions were either unimodal (visual-only or auditory-only) or bimodal (visual + auditory), with participants instructed to attend and respond to only one modality while ignoring the other. In bimodal conditions, participants were explicitly told which modality to attend to before trial onset. Participants were instructed to track the moving target as accurately as possible and, when numbers were presented (visual or auditory), to add them as accurately as possible: neither task was framed as primary. They were further told that if they made a mistake during addition they should not abandon the running total, as the final reported sum would deviate less from the true value if they continued than if they stopped. We therefore made no attempt to dictate an internal prioritisation strategy beyond asking participants to perform both tasks as well as they could.

Each trial began with the target positioned at 270° on the horizontal meridian. Following a drift correction at this initial position, participants were informed about the task condition (control, bimodal congruent, unimodal, or bimodal incongruent) and, where relevant, the target modality (visual or auditory). The pursuit task lasted approximately 30 s per trial, after which the stimulus disappeared and participants verbally reported the final sum, which was entered by the experimenter via keyboard.

Given the increased complexity of the bimodal conditions in comparison to our previous work (Kaye et al., 2025), all participants were given the opportunity to initially complete a practice block in which each of the six conditions was presented once, with pursuit duration reduced to 10 s. Following practice, participants completed the main experimental block consisting of 18 trials (3 repetitions per condition), presented in pseudorandomised order.

At the completion of the smooth pursuit task, participants were required to complete three working memory tasks from the Cognitive Demand Battery (Kennedy et al., 2008) to assess individual WM capacity. The first two tasks were serial subtraction tasks in which participants were given a randomly generated three-digit number (between 900 and 999) and instructed to subtract either 3 or 7 repeatedly for two minutes, entering responses via on-screen digit buttons. Performance on each serial subtraction task was scored as the number of correct subtractions produced within the two-minute window, and this served as the Serial-3 and Serial-7 index in the working-memory composite (see Data analysis). Calculation error was computed by comparing the participant’s final reported sum against the objectively correct running total. That is, the correct sum was determined by cumulatively adding each presented digit in sequence; the participant’s verbal response at the end of the trial was then compared against this value. If participants made intermediate errors (e.g., misadding a digit partway through), these propagated through the running total, so the error metric reflects only the discrepancy between the final reported value and the true cumulative sum. The third task was a Rapid Visual Information Processing (RVIP) task in which participants monitored a continuous stream of single digits (1–9) presented at 100 ms per digit and pressed the spacebar whenever they detected three consecutive even or three consecutive odd digits. The digit sequence was generated to contain approximately 8% targets, with inter-target distances randomly selected between 5 and 20 digits. All three tasks were administered using the standardised Inquisit implementation of the Cognitive Demand Battery.

### Data processing

EyeLink data files were converted from EDF to ASC format and imported into R via the ‘read.asc’ function from the ‘eyelinker’ package. We extracted eye position data from the entire tracking period following target motion onset. Blinks detected by the EyeLink 1000 Plus were removed during initial preprocessing. All trials then underwent visual inspection to identify and remove any additional artifacts in the XY coordinate data that may have been missed by the automated detection algorithm.

Trials were excluded if participants failed to maintain tracking of the moving target. Our exclusion criteria monitored multiple indicators of poor data quality, including prolonged blinks, excessive saccadic activity, and extended periods of stationary gaze. In practice, we applied two specific thresholds: trials were removed if more than 30% of data was lost to blinks or artifacts, or if the XY coordinate pattern failed to show the sinusoidal structure characteristic of circular tracking. Based on these criteria, 6 trials were excluded for excessive data loss and 7 for poor tracking quality or other issues, representing 1.81% of the total 720 trials. The distribution of excluded trials across conditions showed no significant difference (χ² = 7.91, df = 5, *p* =.161). To ensure full engagement with the tracking task, the initial 2 s of each trial were excluded from analysis.

As a complementary index of cognitive demand, we examined blink-related data loss across conditions using a zero-inflated beta regression model to accommodate the point mass at zero (approximately 17% of trials contained no blinks). Estimated blink proportions were highest in the bimodal incongruent auditory (≈ 5.1%) and unimodal auditory (≈ 4.7%) conditions, and lowest in the control condition (≈ 3.2%), with visual-attention conditions falling in between (unimodal visual ≈ 3.8%; bimodal incongruent visual ≈ 4.1%; bimodal congruent ≈ 4.5%). This gradient is consistent with evidence that blink rate increases under greater cognitive demand (Magliacano et al., 2020; Siegle et al., 2008) and with the significant positive relationship between blink-related data loss and radial variability reported in our previous study using the same paradigm (Kaye et al., 2025). Importantly, blink proportion was included as a grand-mean-centred covariate in all pursuit variability models, ensuring that the modality asymmetry in radial variability is not an artefact of differential data loss.

Consistent with our previous approach (Kaye et al., 2025), saccades were retained in the smooth pursuit data rather than removed prior to analysis. Although saccades might be viewed as noise distinct from smooth pursuit behaviour, we treated them as integral to the overall tracking strategy, with their characteristics potentially revealing important aspects of oculomotor responses to task demands. This decision aligns with contemporary theoretical perspectives suggesting that saccadic and pursuit behaviours represent coupled outputs of a unified sensorimotor process (Goettker & Gegenfurtner, 2021; Orban de Xivry & Lefevre, 2007). All analyses therefore included both smooth pursuit and any embedded saccadic movements.

We chose an analytical approach which prioritised trajectory consistency over precise phase alignment between eye and target position. As we previously noted (Kaye et al., 2025), although phase shift corrections can account for temporal offsets between eye and target (Stubbs et al., 2018), radial variability measures yield comparable patterns without such adjustments. Furthermore, tangential error metrics that incorporate phase shift may be artificially elevated by blinks. When participants blink more frequently in certain conditions, they often resume tracking at a displaced position, requiring time to reacquire the target. The resulting catch-up movements inflate tangential variability in ways that reflect momentary visual interruption rather than underlying control deficits.

We therefore focused on tracking stability over time, specifically measuring how consistently gaze maintained its radial distance from the target throughout pursuit. Radial standard deviation served as our primary dependent measure, quantifying tracking stability independent of angular displacement. This metric captures eye movement consistency without confounding effects from minor phase lags or brief catch-up saccades. As a complementary index of task difficulty, we also measured the proportion of data excluded due to blinks within each trial, given established links between blink rate and cognitive demands (Kaye et al., 2025; Magliacano et al., 2020; Siegle et al., 2008).

### Data analysis

We analysed smooth-pursuit variability (radial SD) and dual-task calculation error in a six-level within-participant design (control, unimodal visual, unimodal auditory, bimodal congruent, bimodal incongruent with attention to the visual stream, bimodal incongruent with attention to the auditory stream). Trials with excessive missing gaze samples were excluded using a threshold criterion (blink proportion ≤.30). For participant-level covariates, we formed a working-memory (WM) composite by z-scoring Serial-3, Serial-7, and RVIP d’ (computed from hits, false alarms, total targets and trials) and weighting them using the first principal component from a principal component analysis (PCA): higher values indicate better WM. Serial-3 and Serial-7 were scored as the number of correct subtractions produced within the two-minute window, and RVIP d’ was calculated with a log-linear correction to avoid infinite values at ceiling or floor (Hautus, 1995). The sign of PC1 was flipped where needed so that all three indices loaded positively, and the composite was itself z-scored for use in the models (M = 0, SD = 1 by construction). Blink proportion (grand-mean centred) was included as a nuisance regressor where indicated.

Analyses were conducted with hierarchical Bayesian regression in brms using the cmdstanr backend. For radial variability, we used a lognormal likelihood to respect positivity and right-skew (Muth et al., 2018; Veenman et al., 2024). Condition was coded with a cell-means parameterisation (0 + condition), with uncorrelated varying intercepts and slopes by participant (1 + condition | ID). We fitted a condition-only model, a model adding blink proportion, and a model adding the condition × WM interaction. Priors were weakly informative and anchored to the data scale via prior predictive checks (Sarma & Kay, 2020; Veenman et al., 2024). Specifically, normal priors on the log-scale condition means were centred near log(median(radial variability) × 1.10), Exponential priors were placed on random-effect SDs, LKJ(2) on correlation matrices, and Normal(0.25, 0.08), lower-bounded at 0, on the residual log-SD. Prior predictive checks confirmed plausible ranges without over-constraining the tails.

For calculation error, defined as “Participant Answer” minus “Correct Sum”, we modelled the remaining (non-control) trials with a hurdle-gamma likelihood to account for a genuine mass at zero (correct responses) and a positive, skewed continuous part. Both the positive-mean (µ) and hurdle (hu) components used cell-means for condition. To improve identifiability, the µ part had uncorrelated varying intercepts and slopes (1 + condition || ID) whereas the hu part had a varying intercept (1 | ID). Priors were again anchored by prior predictive checks: normals on log(µ) centred near log(median non-zero error × 1.10) with moderate SD, Exponential priors on µ and hu random-effect SDs, Gamma(4, 1) on the gamma shape, and normals on logit(hu) centred at the observed overall zero rate. As a sensitivity analysis we also fitted a Student-t model to error with the same fixed-effects but correlated varying intercepts and slopes (1 + condition | ID).

All models were run with 4 chains, 5,000 iterations (1,000 warm-up), adapt_delta = 0.98, and max_treedepth = 14. Convergence and sampling quality were assessed via divergences, R̂, and effective sample sizes. Posterior predictive checks (overall, by condition, and zero proportions) were examined for fit (Van de Schoot & Miočević, 2020; Veen & Egberts, 2020). We compared models using leave-one-out cross-validation via PSIS-LOO (Vehtari et al., 2017), which estimates how well each model predicts new data by iteratively holding out observations and assessing prediction accuracy. Models were then weighted using stacking (Yao et al., 2018), where stacking weights represent each model’s optimal contribution to combined predictions—higher values indicate better predictive performance. ΔELPD (difference in expected log predictive density) quantifies pairwise differences in predictive accuracy, with |ΔELPD/SE| > 4 considered substantial. Pareto-k diagnostics were used to identify influential observations (k > 0.7), which were handled via moment matching when detected. For inference, we report estimated marginal means and pairwise contrasts via emmeans. For the hurdle-gamma model, results are on the response scale and thus represent the unconditional mean E(Y) (combining the hurdle and positive parts). Credible intervals are 95% highest-posterior-density intervals unless noted (Phelan et al., 2019).

## Results

Our analysis revealed three main patterns. First, pursuit was more stable when participants attended to visual versus auditory information. Second, adding conflicting cross-modal information increased cognitive errors but did not further destabilise pursuit, suggesting executive control prioritises motor stability when resources are taxed. Third, working memory capacity showed a modest protective effect on pursuit variability, particularly under auditory attention conditions, though this pattern had substantial statistical uncertainty.

### Pursuit variability

**Fig. 2:**
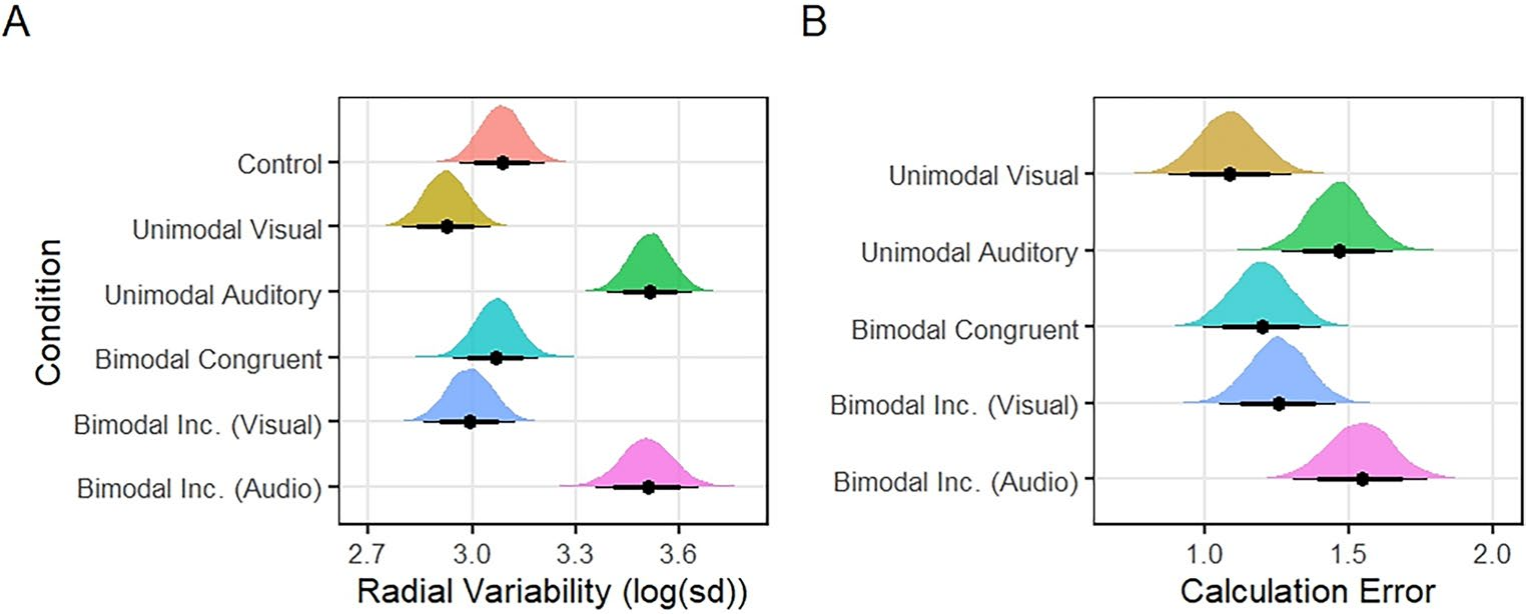
Posterior distributions of estimated marginal means by condition. **A** Predicted Radial Variability (log(sd)) from the lognormal model. **B** Predicted Calculation Error from the hurdle-gamma model. Note that the control condition had no concurrent secondary task, so no errors can be plotted. Dots indicate posterior medians; thick and thin lines represent 80% and 95% credible intervals, respectively

A clear modality asymmetry emerged: conditions emphasising visual information yielded lower radial variability than those emphasising auditory information (Fig. 2A). On the log scale, unimodal visual tracking was credibly more stable than unimodal auditory tracking (median difference in predicted radial SD = − 0.59, 95% CrI [− 0.69, − 0.49]). This corresponds to a multiplicative ratio of 0.55 [0.50, 0.61], indicating that visual attention reduced pursuit variability to 55% of that observed under auditory attention—a 45% reduction (95% CrI [39%, 50%]). A similar advantage was observed when visual information had to be ignored: unimodal visual remained lower than incongruent audio (log-difference = − 0.58 [− 0.73, − 0.43], ratio = 0.56, representing a 44% reduction). By contrast, unimodal auditory and incongruent audio did not differ (log-difference = 0.01 [− 0.12, 0.14], ratio = 1.01 [0.89, 1.15]), suggesting that adding incongruent visual input does not appreciably increase pursuit variability once auditory attention is engaged. Given that the step from control to unimodal auditory produced a 35% increase in radial variability, and the credible interval on this further step excludes any increase larger than approximately 15%, we read this contrast as evidence for a bound rather than a failure to detect a difference. This pattern is consistent with a saturation ceiling on oculomotor disruption: once auditory attention displaces visual support, the extent of destabilisation appears to be set by architectural constraints rather than by cognitive load per se.

The control condition (no secondary task) also showed lower variability than unimodal auditory (log-difference = − 0.43 [− 0.52, − 0.33], ratio = 0.65 [0.59, 0.72], a 35% reduction), confirming that the auditory dual-task condition disrupts pursuit relative to undivided tracking. Importantly, the control condition showed credibly higher variability than the unimodal visual condition (log-difference = 0.17 [0.06, 0.27], ratio = 1.18 [1.06, 1.31]), indicating that visual dual-task demands improved pursuit. This bidirectional pattern demonstrates that the modality asymmetry cannot be attributed to general dual-task costs: visual and auditory secondary tasks push pursuit variability in opposite directions relative to the control baseline. The cost is specific to auditory attention, whilst visual attention, which engages the same sensorimotor circuits that support pursuit, produces a net stabilisation. Model comparison strongly supported the condition × WM interaction model (including blink as a covariate), which received a stacking weight of 0.81, indicating it would contribute 81% to optimal predictions and substantially outperformed simpler alternatives (direct model: 0.12; null model: 0.07). Pareto-k diagnostics identified one influential observation in the WM model (k ≈ 0.89), which was corrected by moment matching; all other models had k < 0.7, indicating reliable inference.

After insightful comments from a reviewer, we also analysed the number of saccades participants performed in each condition. Saccade counts were modelled using a Bayesian negative binomial mixed-effects model with condition as a fixed effect and by-participant random intercepts and slopes. The model converged well (Rhat = 1.00 for all parameters; adequate bulk and tail ESS). Posterior estimates indicated credible differences in saccade count across conditions. The unimodal auditory condition produced the highest predicted counts (exp(3.18) ≈ 24.0), while unimodal visual produced the lowest (exp(2.66) ≈ 14.3). Pairwise contrasts revealed that unimodal auditory was associated with credibly more saccades than both unimodal visual (ratio = 1.68, 95% HPD [1.52, 1.86]) and bimodal congruent (ratio = 1.43, 95% HPD [1.31, 1.56]), with HPD intervals excluding 1.0. The bimodal conditions fell between the two unimodal extremes, and the control condition was broadly intermediate. This pattern of results is consistent with our expectations as saccadic and pursuit behaviours likely represent coupled outputs of a unified sensorimotor process. However, it seems important to determine if our pattern of results is driven exclusively by saccades or variability in smooth pursuit. To this end, we fitted a new model in which grand-mean centred saccade count was added as a fixed covariate to the radial variability model. The results showed that saccade count was a credible predictor of radial SD, with each additional saccade associated with a 2.2% increase in radial variability [95% CrI: 1.8%, 2.7%]. Nevertheless, the condition effect was not eliminated: the rank ordering of conditions was fully preserved and coefficients shifted by no more than 0.11 log-units relative to the model without the saccade covariate, suggesting that saccadic activity partially but did not fully account for the condition differences in radial variability.

Finally, to assess whether modality conditions differentially affected temporal coupling between eye and target, we examined phase relationships using a Bayesian Gaussian mixed model. Tracking was uniformly reactive across all conditions (posterior median phase lag: −0.21° to − 0.25°; posterior probability of lagging > 0.99), with no meaningful differences across conditions (all pairwise contrasts < 0.04°, all credible intervals spanning zero), indicating that the modality asymmetry in radial variability is not driven by differential temporal prediction across conditions.

### Dual task calculation performance

On the response scale under a hurdle-gamma model, posterior estimates indicated that unimodal visual had the lowest arithmetic error, whereas bimodal incongruent with auditory attention tended to be highest (Fig. 2B). The unimodal visual condition produced 37% lower error than bimodal incongruent auditory (ratio = 0.64, 95% HPD [0.46, 0.84]), and 31% lower error than unimodal auditory (ratio = 0.69 [0.53, 0.87]), both providing clear evidence that visual attention supports better calculation accuracy. Error in the unimodal auditory condition was probably lower than in the bimodal incongruent auditory condition, but the evidence was weak (ratio = 0.93 [0.67, 1.19]), as the credible interval included no difference (1.0). Model comparison strongly favoured the hurdle- gamma specification over a Student-t alternative (ΔELPD ≈ + 163.8 ± 16.3), consistent with a zero-generating process in which many participants answered perfectly whilst others made errors of varying magnitude. Posterior predictive checks for the overall and condition-wise zero proportions confirmed excellent model fit.

### Working memory moderation

**Fig. 3:**
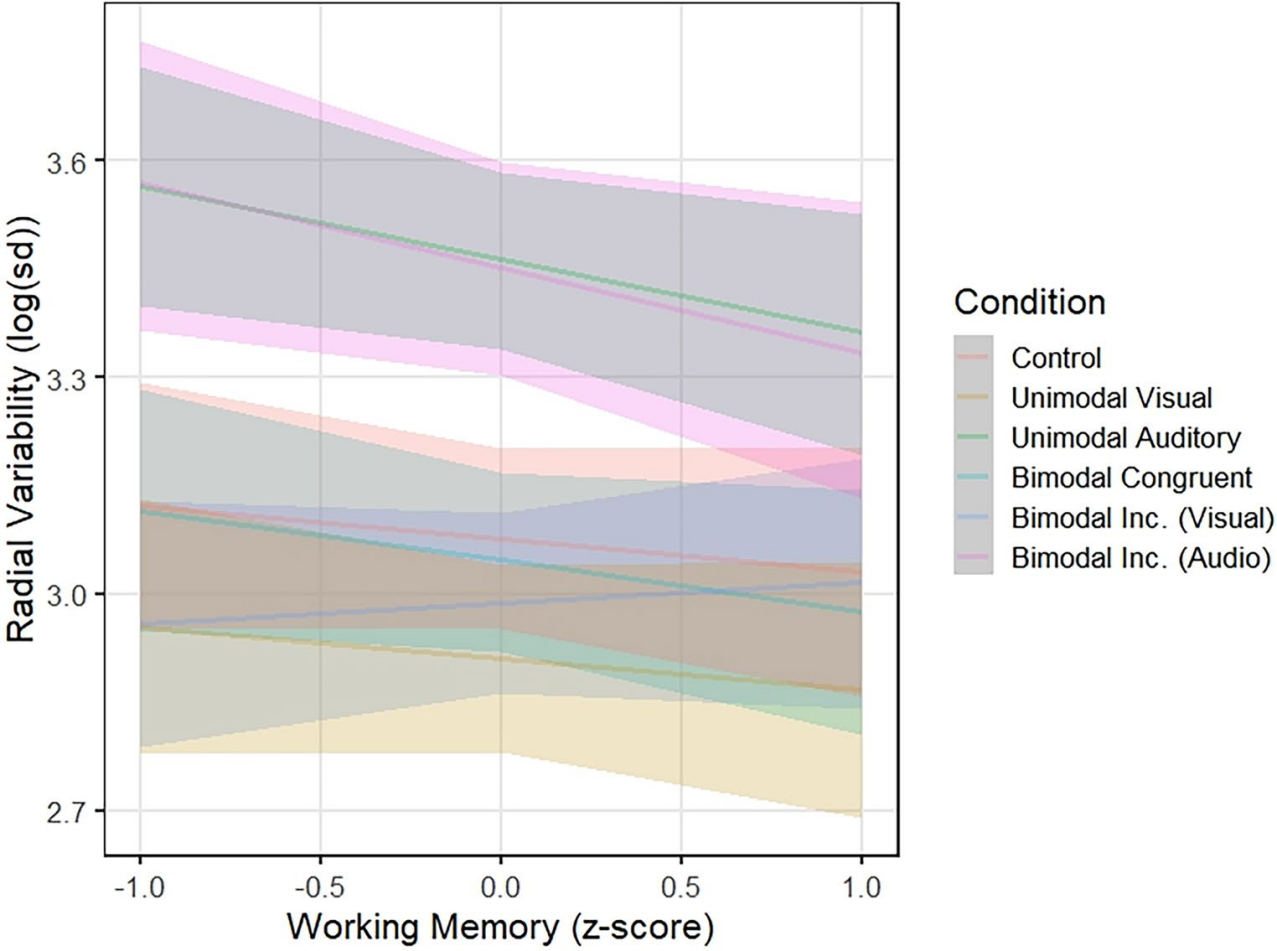
Moderating effect of Working Memory (WM) on pursuit variability. Predicted radial variability (log(sd)) is plotted as a function of continuous WM (z-score) for each condition. Lines represent posterior medians; shaded ribbons represent 95% credible intervals

The condition × WM interaction model (including blink proportion as a covariate) suggested a modest buffering effect of WM that was most pronounced in auditory-attention conditions (Fig. 3). WM slopes on the log scale were negative in unimodal auditory (β = −0.10, 95% CrI [− 0.21, 0.02]) and bimodal incongruent auditory (β = −0.12 [− 0.26, 0.02]), corresponding to 9.5% and 11.3% reductions in radial variability per + 1 SD increase in WM, respectively. Effects in other conditions were smaller: control (β = −0.05 [− 0.16, 0.07], 4.9% reduction), unimodal visual (β = −0.04 [− 0.16, 0.09], 3.9% reduction), and bimodal congruent (β = −0.07 [− 0.19, 0.04], 6.8% reduction). The bimodal incongruent visual condition showed a small increase (β = 0.03 [− 0.09, 0.15], 3.0% increase). To better understand the auditory buffering pattern: imagine two participants, one with low WM capacity (1 SD below average) and one with high WM capacity (1 SD above average). Under unimodal auditory conditions, the high-WM participant would be expected to show approximately 18% less pursuit variability (combining ± 1 SD effects: 1.095² − 1 ≈ 0.20). However, the wide credible intervals, which include zero for all conditions, indicate substantial uncertainty about these estimates. Formal model comparison using PSIS-LOO showed only trivial, statistically indistinguishable differences for the interaction model over simpler alternatives (WM interaction vs. direct model: ΔELPD = − 0.70 ± 3.76; WM interaction vs. total model: ΔELPD = − 0.45 ± 3.78). This suggests the data do not strongly favour including the WM × condition interaction despite its theoretical motivation. A separate WM-only model, controlling for blink but omitting the condition interaction, showed no predictive gain over a blink-only model (ΔELPD ≈ − 1.51 ± 2.94). Across all conditions, the overall WM effect in this model corresponded to a 6.8% reduction in radial variability per + 1 SD of WM ability (95% CrI [− 15.1%, + 4.7%]). The posterior probability that this effect is truly negative was approximately 0.88 (evidence ratio ≈ 7.5), suggestive of a WM-related reduction in variability but not strong enough to be conclusive.

For calculation error, the estimated WM effect was smaller and more uncertain. Working memory capacity was associated with a median 3.2% reduction in errors per + 1 SD (95% CrI [− 15.4%, + 10.3%]), and the posterior probability of a negative effect was only approximately 0.69 (evidence ratio ≈ 2.2). This points to a weak buffering influence of WM on calculation errors, markedly less than that observed for pursuit variability. This dissociation suggests that when cognitive resources are taxed, individuals with higher WM capacity may strategically protect motor performance at the expense of concurrent cognitive tasks.

## Discussion

The primary goal of the present study was to examine how SPEM are influenced by concurrent cognitive demands that vary in sensory modality and cross-modal conflict. We additionally explored whether individual differences in working memory (WM) capacity moderated these effects. In our recent work (Kaye et al., 2025), we demonstrated a modality-specific asymmetry: a secondary visual task reduced SPEM variability, whereas an auditory task of equivalent difficulty increased it. These findings suggested that pursuit interference depends not simply on cognitive load per se, but on the alignment between sensory modality and the attentional resources that support oculomotor control. However, that study employed relatively simpler dual-task conditions in which numbers were presented in a single modality at a time. It remained unclear whether these effects would persist under cross-modal conflict, where competing information must be actively filtered, and whether individuals with greater WM capacity might be buffered against such interference. The present study addressed these gaps by introducing bimodal conditions with congruent and incongruent digit streams whilst assessing individual differences in WM capacity through standardised cognitive tasks. Our results revealed three key patterns: first, we replicated and extended the bidirectional modality asymmetry first reported in Kaye et al. (2025): visual attention stabilised pursuit beyond control levels (ratio = 1.18 [1.06, 1.31]), whilst auditory attention substantially destabilised it (ratio = 0.65 [0.59, 0.72]). This pattern held across both unimodal and bimodal conditions. Second, when attention was directed to an auditory stream in the presence of incongruent visual information, pursuit variability did not increase further; instead, the additional conflict appeared to burden cognitive performance, with a tendency towards increased calculation error. Third, WM capacity offered a modest but statistically uncertain buffer— credible intervals spanning zero, reducing pursuit variability primarily under auditory conditions. We interpret these findings as evidence that dual-task costs in SPEM depend critically on whether attentional selection is representationally aligned with the sensorimotor circuits that drive pursuit: a principle we term Selection-with-Support (SWS).

### Selection-with-Support: an organising principle

To understand why sensory modality exerts these effects on pursuit, we propose Selection-with-Support (SWS) as an organising principle. SWS proposes that dual-task interference in pursuit is minimised when attentional selection is supported by the same representational and neural systems that control the movement and is maximised when selection must be maintained in a format orthogonal to those systems. This principle draws on premotor theories of attention, which propose that covert spatial attention is closely tied to the preparation of eye movements within oculomotor circuits (Buschman & Kastner, 2015; Rizzolatti et al., 1987, 1994; Sheliga et al., 1995). According to this view, directing visual attention to a moving target is not simply a matter of allocating cognitive resources, it is an act of motor planning that directly engages the oculomotor system. Extensive evidence demonstrates that visual selective attention and pursuit control share neural substrates within a visuomotor network encompassing the frontal eye fields (FEF), supplementary eye fields (SEF), parietal cortex, and visual cortex (Gehmacher et al., 2024; Souto & Kerzel, 2021). Neuroimaging studies show that the medial FEF exhibits increased activity during cognitively demanding visual pursuit tasks (Schroder et al., 2023), whilst transcranial magnetic stimulation of the frontal pursuit area affects both pursuit gain and concurrent attentional performance in a correlated manner (Jin et al., 2021). Under these conditions of representational alignment, what we term “supported selection”, the oculomotor and attentional systems work in tandem, requiring minimal executive oversight.

In contrast, auditory attention lacks this premotor grounding. When participants must sustain attention on an auditory stream whilst tracking a visual target, the attentional selection process operates in a format orthogonal to visuomotor circuits. This mismatch necessitates top-down executive control to bridge the representational gap, diverting prefrontal resources that would otherwise support predictive and corrective pursuit mechanisms. This executive bridging may involve generating internal visual representations from auditory input, a form of mental imagery that creates additional temporal and spatial demands distinct from direct visual perception (Gurtner et al., 2021). In fact, Korda et al. (2023) demonstrated that internal visuospatial tasks, akin to imagery, increase pursuit variability even more than arithmetic tasks. This finding is consistent with the hypothesis we previously put forward (Kaye et al., 2025) suggesting that auditory tasks may destabilise pursuit because they require the generation of internal visual representations that compete for shared resources. Complicating matters further, auditory and visual processing are deeply intertwined through cross-modal interactions: spoken language can activate primary visual cortex (Seydell-Greenwald et al., 2023), whilst processing visually presented letters appears to recruit auditory association areas (BA 21/22), suggesting a substrate for phonological recoding (Kirschen et al., 2010). This interaction can be characterised as a “push-pull” mechanism, where high perceptual load in one modality (e.g., visual) can momentarily deplete shared capacity, reducing neural responses in the other (e.g., auditory) (Molloy et al., 2015). Whilst this accounts for general interference, it does not fully explain the directional asymmetry we observed. Critically, however, the mere presence of cross-modal cortical activation does not eliminate the representational asymmetry: whilst both modalities may recruit both sensory cortices, the control architecture differs. Visual input can directly drive oculomotor planning through feedforward visuomotor pathways, whereas auditory input requires active executive transformation to guide a visual-motor task. Thus, whilst shared capacity limits exist, SWS specifies that the cost of this sharing is determined by motor grounding: visual attention engages the very sensorimotor loops required for pursuit, whereas auditory attention lacks this premotor alignment. It is this architectural constraint, not simply which cortical areas are active, that creates the modality asymmetry we observe. These cross-modal demands may further burden the oculomotor system during “unsupported selection,” when attention must be sustained in a modality orthogonal to the motor task. The SWS framework thus provides a mechanistic account of when and why modality matters: not merely because different sensory channels are engaged, but because of the presence or absence of direct motor grounding.

We should note that the characterisation of auditory attention as lacking premotor grounding does not imply that auditory processing is devoid of motor involvement altogether. A growing body of evidence demonstrates motor contributions to auditory attention, particularly in the temporal domain. For example, Morillon et al. (2015) argued that the motor system actively samples auditory input through rhythmic oscillatory mechanisms, providing temporal priors for auditory cortex, and Bengtsson et al. (2009) demonstrated that auditory rhythm processing engages premotor and supplementary motor cortices. These findings establish that auditory attention can recruit motor systems. However, the motor circuits involved are those supporting temporal prediction and rhythmic entrainment, not the visuospatial oculomotor circuits that drive smooth pursuit. In this context, the SWS principle we put forward does not claim that auditory attention is non-motoric in general; rather, it proposes that the specific motor systems engaged by auditory attention in our task are not those that support pursuit control. Pursuit depends on continuous visuospatial transformation within oculomotor circuits (FEF, SEF, parietal cortex), and it is the alignment between attentional selection and these particular circuits that determines dual-task cost. Auditory-motor coupling in the temporal domain may well support other forms of sensorimotor coordination (e.g., musical performance see Chen et al. (2009) without providing the spatial-motor grounding that pursuit requires.

### Modality asymmetry and cross-modal conflict

The present findings extend our understanding of supported versus unsupported selection in two important ways. First, the bidirectional asymmetry persisted even under bimodal conditions requiring active filtering of incongruent information. Visual attention continued to stabilise pursuit, whilst auditory attention continued to destabilise it, regardless of whether conflicting cross-modal information was present. This demonstrates that the stabilising effect of visual attention is not simply a product of sensory channel preference but reflects genuine representational alignment: tracking a target whilst attending to visual digits embedded within it allows the attentional and motor systems to operate in concert, producing tracking that is more stable than undivided pursuit alone. Conversely, attending to auditory information failed to stabilise pursuit even when irrelevant visual distractors were present, confirming that the disruptive effect stems from a fundamental architectural constraint rather than mere distraction.

Second, adding cross-modal conflict to unsupported selection did not further degrade pursuit. When participants attended to auditory digits whilst ignoring incongruent visual digits, pursuit variability remained comparable to the unimodal auditory condition (Fig. 2A). Instead, the cost of incongruence manifested primarily as a tendency towards increased arithmetic errors (Fig. 2B), though the evidence for this difference was weak (95% highest posterior density interval included zero; posterior predictive checks suggested approximately 85% probability that bimodal-incongruent-auditory error exceeded unimodal-auditory error). This dissociation between pursuit stability and cognitive performance suggests two complementary mechanisms. First, pursuit destabilisation may reach a saturation ceiling on oculomotor disruption once auditory attention is engaged, consistent with evidence that spatial auditory tasks co-opt visuomotor control networks like the dorsal attention network (Braga et al., 2016). Within this constraint, however, executive control systems appear to actively prioritise motor stability when additional conflict arises. Rather than allowing pursuit to degrade further (which it largely cannot, given the saturation ceiling on oculomotor disruption), the system absorbs the additional conflict cost cognitively, protecting whatever oculomotor consistency remains. This prioritisation reflects competition for complementary frontal networks specialised for spatial-motor versus nonspatial auditory processing (Fougnie et al., 2018; Michalka et al., 2015). This pattern mirrors recent findings in the auditory domain (Gustafson et al., 2023), where listeners maintaining speech recognition performance in high-load noise conditions nonetheless exhibited significantly longer verbal response times, indicating a spike in listening effort. Just as those listeners maintained primary task performance at the cost of processing efficiency, our participants may have maintained pursuit stability at the cost of cognitive accuracy. Under high load, task prioritisation can shift dynamically depending on which system is already under strain (Wickens, 2002, 2008), and here the system appears to maintain pursuit at the expense of some calculation accuracy.

### Working memory as possible selective buffer: exploratory evidence

Individual differences in WM capacity moderated pursuit variability in a pattern consistent with SWS, though the statistical evidence was modest. Participants with higher WM showed approximately 9.5% and 11.3% reductions in pursuit variability per standard deviation increase in WM for unimodal auditory and bimodal incongruent auditory conditions, respectively, compared to smaller or near-zero effects in visual conditions. However, 95% credible intervals overlapped zero, and formal model comparison via PSIS-LOO revealed only trivial differences between models with and without the WM interaction. Thus, whilst the qualitative pattern converges with theoretical expectations, the statistical support is suggestive rather than conclusive.

Despite the uncertainty in our estimates, the direction of these effects is at least consistent with a selective buffering account within the SWS framework. When selection is supported by visual input hypothesised to engage oculomotor circuits via shared sensorimotor substrates, executive demands are minimal, and individual differences in WM capacity confer little additional advantage (Pierrot-Deseilligny et al., 2005). Under unsupported selection, however, sustaining auditory attention whilst coordinating pursuit requires greater executive effort. Individuals with higher WM capacity may therefore possess a modest reserve of executive resources deployable to stabilise pursuit when attentional selection lacks motor grounding (Vogel et al., 2005). This interpretation aligns with evidence that maintenance of auditory-verbal information recruits dorsolateral prefrontal cortex, which also participates in executive control of eye movements (Funahashi & Andreau, 2013; Tachibana et al., 2012). Notably, the WM effect on calculation errors was even weaker and more uncertain (median − 3.2%, 95% credible interval − 15.4% to + 10.3%), suggesting that WM capacity may offer more protection to the motor system than to the concurrent cognitive task. This is a pattern consistent with theories proposing that high cognitive loads force strategic prioritisation whereby remaining resources stabilise the primary motor task at the expense of the secondary cognitive one (Beilock & Carr, 2005). Future studies seeking to confirm WM-mediated buffering in smooth pursuit should employ adequately powered designs based on our effect estimates, as the theoretical considerations outlined above, together with the directional patterns observed here, provide a motivation for such work.

### Theoretical integration

How does SWS relate to established theories of attention and dual-task performance? Classical resource-based accounts, such as Multiple Resource Theory (Wickens, 2002, 2008), predict that tasks drawing on separate sensory modalities should interfere less than tasks competing within the same modality. Whilst our findings partially align with this prediction, the resource model alone cannot explain why auditory attention imposed substantially greater costs on pursuit than visual attention, despite both involving distinct sensory channels. Similarly, the Time-Based Resource-Sharing model (Barrouillet & Camos, 2014; Glavan & Houpt, 2019) proposes that interference stems from temporal competition for a single attentional bottleneck. Yet our results show that attending to auditory versus visual information produced asymmetric costs despite comparable cognitive demands, suggesting that interference depends not merely on temporal resource-sharing but on the specific modality being maintained.

Importantly, SWS can be distinguished from these standard resource accounts by proposing that the impact of a secondary task depends on its representational nature rather than just its difficulty or modality. This distinction is supported by evidence that working memory load is not a unitary construct. For example, (Konstantinou et al., 2014) demonstrated that whilst loading executive control increases distractibility, loading visual maintenance actually reduces it by recruiting sensory cortices involved in perception. SWS extends this dissociation to the oculomotor domain: visual secondary tasks act akin to visual maintenance, recruiting premotor circuits that “support” pursuit and shield it from interference, whereas auditory tasks function like executive load, requiring a resource-draining bridge between orthogonal modalities.

This representational view aligns well with integrated architectures like the Working Memory with Distributed Executive Control (WMDEC) model (Vandierendonck, 2021), which proposes distinct modules including modality-specific buffers and a central executive that manages goal-directed action. From this perspective, the modality asymmetry we observed reflects differences in dual-task coordination costs: the cost is minimal when visual attention guides a visual-motor task, as components map onto aligned systems, but increases substantially when auditory information must guide a visual-motor task, requiring effortful executive coordination across separate representations. SWS complements this view by specifying the mechanistic basis for these coordination costs: premotor coupling when attention and action share representational space, and executive bridging when they do not. In this sense, SWS does not replace resource-based or architectural accounts but rather provides a mechanistic bridge that clarifies when and why coordination costs arise. It is consistent with Multiple Resource Theory’s emphasis on modality-specific channels but adds the critical insight that modality matters because of motor grounding, not merely sensory separation. It is consistent with premotor theory’s claim that attention and motor preparation are coupled but extends this by specifying that coupling is conditional on representational alignment and that executive control can partially compensate when alignment is absent.

### Limitations and future directions

Several limitations must be acknowledged here. One consideration is the spatial relationship between the secondary-task stimuli and the pursuit target. In the present design, visual digits were presented within the moving target disc, meaning that visual attention and oculomotor tracking were directed to the same spatial location. Auditory digits, by contrast, were delivered binaurally through headphones and carried no spatially localised information. It is therefore possible that part of the modality asymmetry reflects differential spatial-attention demands: visual conditions permitted unified spatial orienting, whereas auditory conditions may have required participants to divide or decouple spatial attention from the pursuit target. We acknowledged a related possibility in our previous study (Kaye et al., 2025), where we noted that the visual numbers embedded in the pursuit target may have provided a more focal point for tracking. We note, however, that several features of our data argue against a purely spatial account. First, the bimodal incongruent auditory condition, in which spatially localised but task-irrelevant visual digits were also present at the target, did not produce greater pursuit disruption than the unimodal auditory condition, suggesting that the mere availability of spatially co-located visual information does not automatically alleviate the auditory cost. If spatial co-location were the primary driver, one would expect the presence of visual digits at the target to partially offset the auditory cost even when attention is directed to the auditory stream, but this was not observed. We acknowledge, however, that participants were instructed to attend to the auditory stream and ignore the co-located visual digits in this condition, so we cannot fully exclude the possibility that an unsuppressed visual anchor could have conferred some benefit; the absence of any such benefit therefore constrains, rather than completely rules out, a spatial-anchoring contribution. Relatedly, a further observation points in the same direction: the saturation-ceiling pattern described earlier (Fig. 2A) implies that once auditory attention has displaced visual support, little spare oculomotor capacity remains for any co-located visual anchor to be exploited, so the bimodal-incongruent-auditory condition may offer little scope for a spatial benefit to express itself. Second, our SWS account predicts the asymmetry specifically because visual attention engages premotor circuits shared with pursuit, not merely because it is spatially coincident; indeed, the premotor theory of attention posits that spatial orienting is itself a motor act (Rizzolatti et al., 1987), making spatial co-location and motor grounding difficult to fully disentangle within any single paradigm. Nevertheless, future studies could explore designs that decouple spatial co-location from modality, for instance by varying the position of visual digits within the pursuit target disc rather than fixing them at its centre, thereby introducing a degree of spatial uncertainty even within the co-located condition, though care would be needed to avoid eliciting saccades away from the tracking trajectory. Alternatively, spatialising auditory stimuli using head-related transfer functions could test whether providing spatial congruence between the auditory stream and the pursuit target reduces the auditory cost, though the ecological validity of such manipulations during continuous pursuit remains to be established. The WM moderation effect, whilst consistent in direction, was modest and did not decisively improve model fit, suggesting that future research with larger samples or populations characterised by executive dysfunction may offer a better opportunity to detect and characterise these effects with greater precision.

A further consideration is that our WM composite was derived entirely from visually presented tasks (Serial-3, Serial-7, and RVIP), whereas the condition in which WM was hypothesised to act as a selective buffer involved sustained auditory attention; to the extent that WM resources are partially modality-specific (Shah & Miyake, 1996), a composite incorporating an auditory WM measure may provide a more sensitive index of the resources actually recruited under unsupported auditory selection. In addition, our paradigm targeted a specific form of spatially anchored visual tracking with verbal auditory load. Therefore, generalisation to different forms of auditory spatial attention, or clinical groups awaits empirical testing. Relatedly, although our account appeals to internally generated visual imagery as one route by which auditory attention may destabilise pursuit (see also Gurtner et al., 2021; Korda et al., 2023), the present design did not isolate imagery from other executive demands of the auditory task; future work manipulating imagery vividness or using imagery-selective secondary tasks could directly test how strongly imagery alone drives the asymmetry we observe.

We note that calculation error represents the terminal error at the end of a trial, not an online measure of cognitive load during a trial. Therefore, future work should consider continuous accuracy metrics to provide a more sensitive index of online processing load. In addition, the present study did not obtain a measure of arithmetic ability. Future studies could control for this variable to account for individual differences in processing load.

## Conclusion

The present findings demonstrate that smooth pursuit is a cognitively modulated behaviour whose dual-task costs depend critically on representational alignment between attentional selection and sensorimotor control. Visual attention stabilises pursuit, beyond single-task levels, because it is premotorically and representationally aligned with the oculomotor system. Auditory attention destabilises pursuit because it must be sustained through executive-mediated bridging across orthogonal representational formats. When this bridging is further stressed by cross-modal conflict, the cost may emerge primarily as cognitive error rather than further motor disruption, suggesting functional limits in how much the oculomotor system can degrade whilst still maintaining task engagement. Working memory capacity may offer selective, if modest, buffering under unsupported conditions, providing a limited executive reserve when attention and action are not naturally aligned.

## Data Availability

The data that support the findings of this study are available from the corresponding author upon reasonable request.
